# Editorial: Metabolic syndrome in patients with diabetes: identification of biomarkers

**DOI:** 10.3389/fcdhc.2025.1620665

**Published:** 2025-06-12

**Authors:** Surjya Narayan Dash, Mónica Muñoz-Úbeda, Faisal Aziz

**Affiliations:** ^1^ Helsinki Institute of Life Science, Biocenter 2, Viikinkaari, University of Helsinki, Helsinki, Finland; ^2^ Department of Cell Biology, University of Virginia, Charlottesville, VA, United States; ^3^ Physical Chemistry Department, Faculty of Chemistry, University Complutense of Madrid, Madrid, Spain; ^4^ Cardiometabolic Trials Unit, Department of Endocrinology and Diabetology, Medical University of Graz, Graz, Austria

**Keywords:** diabetes, mitochondria, obesity, biomarkers, metabolic syndrome

Diabetes is a prolonged metabolic disease that causes major harm to the nerves, blood vessels, eyes, heart and kidneys. Hyperglycaemia or increase in blood glucose levels are hallmark of diabetes. Type 1 diabetes is a chronic illness in which the pancreas is incapable of producing sufficient insulin on its own, whereas Type 2 diabetes (T2D) is the most prevalent, typically affects adults, and develops due to either insulin insufficiency or resistance. The prevalence of T2D has sharply increased during the last three decades in all nations and age groups. Diabetes is a global pandemic and diabetes-related health expenditures were estimated at 966 billion USD in 2021 and are projected to reach 1,054 billion USD by 2045 (1). According to World Health Organisation (WHO), diabetes affects over 830 million people globally. Over the past few decades, there has been a steady rise in the number of persons with diabetes and those who do not get treatment for the disease. The primary global objectives of researchers are to either stop or limit the increase of diabetes. While the etiology of T2D is many-sided, the induction of insulin resistance (IR) is a key phenomenon, and impairments in insulin signaling directly contribute to hyperglycaemia.

**Figure 1:**
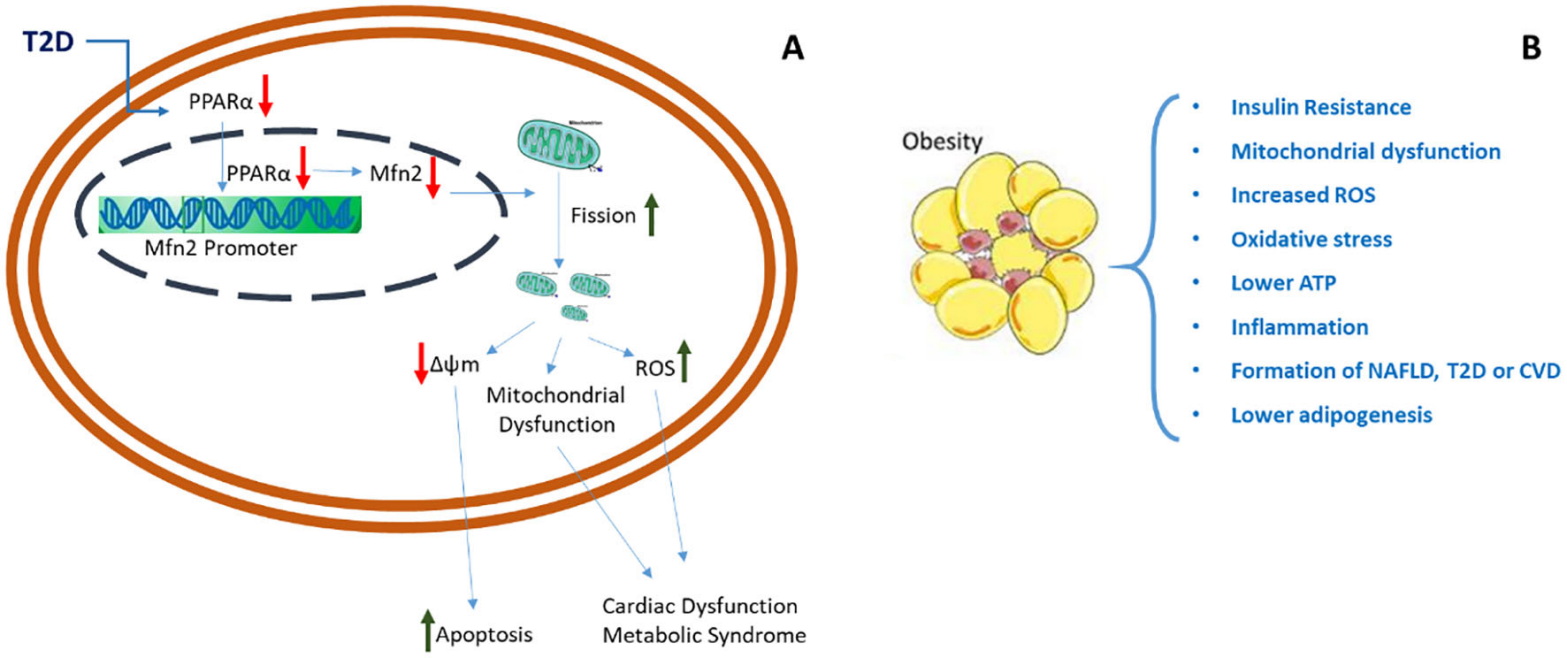
A schematic representation of the underlying causes of both obesity and T2D. **(A)** MetS, cardiac dysfunction, and mitochondrial dysfunction brought on by the onset of T2D. **(B)** The consequences of obesity.

A symptom of IR, hyperinsulinemia is caused by increased β-cell production of insulin and decreased insulin clearance due to downregulated insulin receptors on target cell surfaces. Hyperinsulinemia is an indicator of IR and a predictor of T2D (2). Mitochondrial activity plays a crucial role in glucose metabolism, and changes in mitochondrial function have been connected to IR and diabetes (3). Research has shown that T2D is associated with decreased expression of the nuclear receptor protein peroxisome proliferator-activated receptor alpha (PPARα), a transcription factor that mainly controls inflammation and lipid and glucose metabolism. Mitofusin 2 (Mfn2) protein expression decreases when PPARα is reduced. Mfn2 contributes to the external fusion of the mitochondrial membrane, which causes an increase in fission and disturbs the equilibrium of the mitochondrial fusion/fission process (**Figure 1**). This suggests oxidative stress and Reactive Oxygen Species (ROS) generation, mitochondrial malfunction, and an increase in cellular apoptosis, which lead to metabolic syndrome (MetS). A high lipid environment is tightly linked to several of the suggested mechanisms that result in the attenuation of insulin signalling, including lipid accumulation and the abnormal production of ROS.

Further, Diabetes mellitus is a metabolic disorder and there is a close relationship between diabetes and MetS (4). The onset and advancement of MetS are strongly impacted by biomarkers and mitochondrial dysfunction, the latter of which is linked to IR and oxidative stress. Traditional studies have shown that muscle mitochondrial structure/function loss contribute to lipid-induced IR. High blood pressure, elevated triglycerides (TG), poor HDL cholesterol, abdominal obesity and higher fasting glucose are among the metabolic risk factors that constitute MetS, which raises the risk of T2D. Charkos et al. evaluated the prevalence of MetS in patients with type-2 diabetes mellitus (T2DM) through a comprehensive review and meta-analysis, found that in T2DM patients, the prevalence of MetS was lower percentage in men than women and emphasises how crucial it is to educate T2DM patients to prevent its associated consequences (5). Alawdi et al., investigated the clinical patterns and prevalence of MetS in individuals with T2D, as well as the relationship between MetS and the effectiveness of anti-diabetic medications. Most individuals with T2D did not achieve satisfactory results from pharmacotherapy, therefore, to enhance the results of treatment early preventive and therapeutic measures for MetS were suggested. Using bibliometric and visualisation tools, Wan et al. examined the molecular processes by which exercise affects MetS and identified important research trends and collaboration networks. According to their research, exercise helps in improving cardiovascular health, lowering inflammation and cholesterol buildup, boosting insulin sensitivity, and reversing the effects of a high-fat diet on abdominal obesity. Teimouri et al. compared patients with pre-diabetes to those with normal glucose tolerance to determine the prevalence of pre-diabetes and examined the cardiometabolic risk factors among non-alcoholic fatty liver disease (NAFLD) patients. According to this study, about one-third of patients with NAFLD also had pre-diabetes, indicating that both conditions were predictive of MetS. The main risk factor for T2D is obesity and which plays a key for developing NAFLD (5). There is substantial evidence linking diabetes to NAFLD (6). Additionally, NAFLD increases the risk of T2D by at least three times (7).

Another factor that influences in T2D patients is the inducible enzyme hemeoxygenase-1 (HO-1) breaks down heme into free iron, biliverdin (which changes into bilirubin), and carbon monoxide (CO). It also has cytoprotective, anti-inflammatory, and antioxidant properties and influences immunological responses. It is currently not fully established that association between HO-1 and hyperlipidaemia in pre-diabetic people, although its significance and advantages are well established in diabetic rodent models (Fan et al.). Fan et al. concluded that in overweight pre-diabetic patients, particularly in females, a raised HO-1 level was directly linked to a lower incidence of hyperlipidaemia. Their results shed light on HO-1 mechanism in hyperlipidaemia while also raising the possibility that gender and body weight may have an impact. In pre-diabetic patients, hyperlipidaemia was adversely correlated with elevated HO-1, particularly in females who were overweight.

Biomarkers, make it easier to study the mechanisms underlying disease and to evaluate novel preventive and therapeutic approaches in diabetes and important for the sensitivity and specificity of prediabetes and diabetes prediction. To determine the clinical utility of biomarkers, more comparative research will be needed. Since diabetes can damage multiple organs (such as the heart, kidney, nerve, and eye) to varying degrees within a single person, tissue-specific biomarkers are particularly important to prevent the progression.
